# Awareness, Attitudes, and Barriers Toward Breast Symmetry Procedures Among Women After Breast Reconstruction: A Cross-Sectional Study

**DOI:** 10.3390/jcm15020506

**Published:** 2026-01-08

**Authors:** Saleh Abualhaj, Mosleh M. Abualhaj, Lina Alshadfan, Yasmin Safi, Mu’taz Massad, Osama Shattarah, Yousef Albustanji, Younis Hizzani, Zain aldeen Saleh, Dima Alhawajreh, Ayyub Masoud, Mohd Said Dawod

**Affiliations:** 1 Department of Surgery, King Hussein Cancer Center, Amman 11941, Jordan; 2 Faculty of Medicine, Al-Balqa Applied University, Al-Salt 19117, Jordan; ayyub.md2003@gmail.com; 3 General Surgery Department, Istiqlal Hospital, Amman 11118, Jordan; 4 Department of Networks and Cybersecurity, Al-Ahliyya Amman University, Amman 19111, Jordan; m.abualhaj@ammanu.edu.jo; 5 Department of Pediatrics, Faculty of Medicine, Al Balqa Applied University, Al-Salt 19117, Jordan; lina.shadfan@bau.edu.jo; 6 Faculty of Medicine, The Hashemite University, Zarqa 13133, Jordan; mutaz@hu.edu.jo (M.M.); younishizzani2002@gmail.com (Y.H.); zainsaleh99@gmail.com (Z.a.S.); 7 Department of General Surgery, Faculty of Medicine, Jordan University of Science and Technology, Irbid 22110, Jordan; oshatarah@yahoo.com; 8 Breast Unit, North West Anglia NHS Foundation Trust, Peterborough City Hospital, Peterborough PE3 9GZ, UK; yousef.albustanji@nhs.net; 9 Faculty of Medicine, The University of Jordan, Amman 11942, Jordan; 10 Special Surgery Department, Faculty of Medicine, Mutah University, Al-Karak 61710, Jordan; msd906@gmail.com

**Keywords:** breast reconstruction, contralateral symmetry, breast symmetry procedures, patient-reported outcomes, patient awareness, barriers to surgery, oncoplastic surgery

## Abstract

**Background:** Achieving breast symmetry is an important aesthetic goal following reconstruction post-mastectomy; however, little is known about women’s awareness, attitudes, and barriers regarding Contralateral Breast Symmetry Procedures (CBSP) in Jordan. Objectives: To assess awareness, perceptions, and barriers toward contralateral breast symmetry procedures among breast cancer survivors who underwent reconstruction at King Hussein Cancer Center. **Methods:** A cross-sectional study was conducted from July to Oct 2025 at KHCC, among 314 women diagnosed with breast cancer who had post-breast reconstruction. Data were collected using a structured Arabic questionnaire, which was developed based on existing literature, validated by an expert panel, and piloted on 10 women for clarity and reliability. The final tool demonstrated acceptable internal-consistency (Cronbach’s α = 0.712). The questionnaire captured sociodemographic and clinical data and detailed knowledge, attitudes, and barriers related to CBSP. Descriptive statistics summarized the data. **Results:** Participants’ mean age was 45.8 years; the majority were married (83.8%) and held university degrees (65.6%). Most reconstructions used silicone implants (94.6%). Only 6.4% had undergone CBSP, primarily delayed breast augmentation or mastopexy, with 75% reporting satisfaction. Awareness of CBSP was limited (37.9%), and less than one-third had discussed CBSP options with their surgeon or knew about insurance coverage. While 82.5% valued symmetry for body image, 31.5% viewed it as unnecessary after cancer recovery. Main barriers included satisfaction with current appearance (48.1%), fear of additional surgery (32.2%), financial constraints (37.3%), and lack of physician counseling (27.1%). Trust in medical team recommendations was high (89.2%). **Conclusions:** Contralateral breast symmetry procedures are under-recognized and infrequently pursued, primarily due to limited awareness, financial concerns, and insufficient counseling. Focused education and enhanced surgeon–patient communication are essential to support women’s aesthetic and psychological needs after reconstruction in Jordan.

## 1. Introduction

Breast cancer is the most commonly diagnosed cancer among women worldwide, accounting for approximately 2.3 million new cases and 685,000 deaths in 2020, representing a major public health concern and leading cause of cancer-related morbidity and mortality in women [1]. Advances in early detection and treatment have significantly improved survival rates, resulting in a growing population of breast cancer survivors who face unique challenges related to long-term quality of life, including physical, psychological, and aesthetic outcomes [2,3,4]. In Jordan, breast cancer is the most prevalent cancer among women, representing nearly 36% of all female cancers, with an increasing incidence over the past decade [5]. This rising burden highlights the need for comprehensive survivorship care that addresses both oncologic outcomes and post-treatment quality of life, including breast reconstruction and contralateral breast symmetry procedures [6]. Breast reconstruction following mastectomy has been demonstrated to have positive effects on the psychosocial well-being, body image, and overall quality of life in breast cancer survivors [7,8,9,10]. Symmetry between the reconstructed and contralateral natural breast is a major goal following unilateral mastectomy, which in many cases is achieved through a contralateral breast symmetry procedure such as augmentation, mastopexy, or reduction [11,12].

CBSP can be performed at the same time as the initial reconstruction or can be staged. The decision is based on patient preferences, anatomy, and the oncologic treatment plan [13,14]. Achieving symmetry is usually more difficult after implant-based reconstruction than autologous techniques. This can be related to the inherent shape of implants as well as differences in tissue change over time [13,14].

Breast reconstruction awareness and availability have increased; however, disparities persist in access and utilization, particularly for symmetry procedures. These may be due to sociodemographic factors, insurance factors, providers’ communication practices and patients’ knowledge. Fear of additional surgery, financial cost, and lack of physician recommendation are also noted to be barriers to the uptake of symmetry procedures [15,16].

Despite legislative mandates (such as the Women’s Health and Cancer Rights Act) that support coverage for reconstruction, and greater awareness of its benefits, disparities in access and utilization persist worldwide. In addition, studies have revealed that in countries with established access to reconstruction, rates of utilization are less than optimal, especially among women of low socioeconomic status, women from ethnic minority groups, and those treated in rural or non-academic centers [15].

Evidence is growing that not only are aesthetic and psychosocial outcomes important to patients, but patient-reported outcome instruments such as the BREAST-Q highlight nuanced differences in satisfaction and physical well-being between those who receive contralateral breast symmetry procedures and those who do not. Emerging data also show that symmetry achieved with augmentation is most likely to result in high satisfaction, with other procedures, such as mastopexy or reduction, having lower physical well-being despite similar satisfaction with appearance, emphasizing the importance of appropriate surgical selection and counseling [14].

Crucially, Razdan et al. now suggest that surgeons should be discussing all options for reconstruction and symmetry as part of a shared decision-making process, recognizing the lasting impact of these decisions on long-term survivorship [14]. Nevertheless, persistent system-level barriers, such as hospital-level barriers, provider specialty, and reconstructive service availability, remain the largest contributors to inequities in access to care, with significant differences still found between non-urban centers and the publicly funded health system. Provider- and patient-level barriers, including cultural and information access, also impact patient decision-making [16]. In Middle Eastern cultures, including Jordan, decisions related to breast reconstruction and subsequent symmetry procedures are strongly influenced by sociocultural norms. Stigma surrounding aesthetic procedures may also limit open discussion and awareness about breast refinement options. These cultural dynamics may affect women’s willingness to pursue secondary symmetry procedures, their comfort in discussing aesthetic concerns, and their perceptions of body image after mastectomy and reconstruction [17,18]. Therefore, understanding these contextual factors is essential when evaluating patient attitudes and barriers within this population.

Therefore, efforts to improve access to symmetry procedures justly should include educating physicians and patients, changes to policy regarding coverage and referrals, and reforms to the healthcare system to reduce practical and financial obstacles [16].

This study aims to evaluate the awareness, attitudes, and barriers to contralateral breast symmetry procedures among women who have undergone breast reconstruction at a tertiary cancer center. Specifically, we assess the prevalence of knowledge about symmetry options, patient interest and satisfaction, and the sociodemographic and clinical factors—along with patient-reported barriers—that influence the consideration and uptake of these procedures. The findings are intended to inform strategies for improving patient counseling and the shared decision-making process, addressing unmet needs in breast cancer survivorship care.

## 2. Materials and Methods

### 2.1. Study Design and Setting

This study employed a cross-sectional survey design to assess patient awareness, attitudes, and perceived barriers toward contralateral breast symmetry procedures following mastectomy and reconstruction among breast cancer patients. The study was conducted at King Hussein Cancer Center (KHCC), a leading tertiary cancer center in Amman, Jordan. Data collection was carried out between 5 July 2025 and 1 October 2025.

### 2.2. Participants

The study population included women who had been diagnosed with breast cancer and subsequently undergone mastectomy and breast reconstruction surgery at King Hussein Cancer Center (KHCC). Eligible participants were those who:

- Had undergone mastectomy and subsequent reconstruction surgery between January 2024 and January 2025 at KHCC.
- Were 18 years or older at the time of participation.
- Were able to provide informed consent and participate in a phone-based interview in Arabic or English.

Patients were excluded if they:

- Had recurrent or metastatic breast cancer at the time of data collection.
- Had incomplete or missing contact information.
- Were unable to communicate due to cognitive or language barriers.

### 2.3. Sampling and Recruitment

Eligible patients were identified from the institutional breast reconstruction registry. A convenience sampling method was used. Trained research personnel contacted patients via telephone between July and October 2025 to invite them to participate in a structured follow-up survey. Verbal informed consent was obtained prior to data collection.

The sample size was calculated using the formula for estimating a single population proportion:

N = (Z^2^ × p (1 − p))/d^2^

where:

- Z = 1.96 (for 95% confidence interval);
- p = 0.5 (assumed proportion of awareness to maximize sample size);
- d = 0.056 (margin of error 5.6%).

The minimum required sample size was ~300 participants, allowing for a 5% non-response rate.

### 2.4. Data Collection Instrument

Data were collected using a structured, phone-based questionnaire developed by the research team to assess awareness, attitudes, and barriers toward contralateral breast symmetry procedures. The questionnaire consisted of four domains: sociodemographic and clinical characteristics, awareness of breast symmetry options, attitudes and perceptions toward aesthetic outcomes and body image, and patient- and system-related barriers to symmetry procedures. The survey tool underwent content validation by a panel of five experts in surgical oncology, plastic surgery, and health psychology to ensure clarity, relevance, and cultural appropriateness. A pilot test was conducted on 10 participants who met the inclusion criteria to evaluate comprehensibility and timing. Necessary modifications were made based on their feedback before launching the full study. The questionnaire was administered in Arabic, following standard forward–backward translation procedures to ensure linguistic and conceptual equivalence. Additionally, the internal consistency of the questionnaire was assessed using Cronbach’s alpha, which demonstrated acceptable reliability across the relevant domains (α = 0.712).

### 2.5. Ethical Considerations

Ethical approval was obtained from the Institutional Review Board (IRB) of King Hussein Cancer Center (Approval No: 25 KHCC 161). All participants provided verbal informed consent prior to participation. Confidentiality was maintained throughout the study, and all responses were anonymized and stored in password-protected electronic databases accessible only to the research team.

### 2.6. Data Management and Analysis

Data were entered and analyzed using R version 4.5.1. Descriptive statistics were used to summarize participant characteristics and survey responses. Categorical variables were presented as frequencies and percentages. No subgroup comparisons were performed due to the small number of participants who underwent contralateral breast symmetry procedures, and the analysis is therefore primarily descriptive.

## 3. Results

### 3.1. Sociodemographic and Clinical Characteristics

Out of 364 eligible patients contacted, 314 responded and completed the survey, resulting in a response rate of 86.3%. The mean age was 45.8 years (SD = 8.8), ranging from 22 to 76 years. The majority were married (83.8%), with smaller proportions being single (8.9%), widowed (3.8%), or divorced (3.5%). Regarding educational level, most participants had a university degree or higher (65.6%), while 29.9% had completed secondary education and only 4.5% had primary education or less. Approximately one-third of the participants (32.5%) were employed at the time of the survey. The vast majority resided in urban areas (92.4%), with only 7.6% living in rural areas. The mean BMI was 28.5 kg/m^2^ (SD = 5.8), with values ranging from 17.7 to 36.6. Most participants were non-smokers (70.7%); 17.8% were current smokers and 11.5% were ex-smokers. About one-quarter of the participants (26.8%) reported having chronic diseases. In terms of menopausal status, just over half were postmenopausal (52.2%), while 47.8% were premenopausal (Table 1).

### 3.2. Clinical and Treatment Characteristics

In terms of cancer stage, more than one-third of participants were diagnosed at stage I (37.9%), followed by stage II (36.0%) and stage III (26.1%). Regarding type of mastectomy, the largest proportion underwent skin-sparing mastectomy (SSM) (44.9%), followed by total mastectomy (36.6%), nipple-sparing mastectomy (NSM) (12.7%), and areola-sparing mastectomy (5.7%). The vast majority of participants (94.6%) had reconstruction with silicone implants, while only 4.5% had autologous tissue reconstruction and 1.0% had a combination of both techniques. The mean time since reconstruction was 23.1 months (SD = 21.9), ranging from 1 to 168 months. With respect to adjuvant therapy, more than half of participants received radiotherapy (53.8%), 77.4% received chemotherapy, and 80.9% received hormonal therapy (Table 2).

### 3.3. Contralateral Breast Symmetry Procedures and Future Considerations

Only a small proportion of participants had undergone a symmetry (balancing) procedure (6.4%, n = 20). Among these, breast augmentation was the most common type (40.0%), followed by mastopexy (35.0%) and breast reduction (25.0%). Regarding timing, over half of the symmetry procedures were performed in a delayed setting (55.0%), while 45.0% were performed immediately at the time of reconstruction. The majority of patients who underwent symmetry reported satisfaction with the results (75.0%), while one-quarter (25.0%) were dissatisfied. Among those who had not yet undergone a symmetry procedure, about one-quarter (26.2%) indicated that they were considering it in the future, while half (50.3%) did not intend to pursue it and 23.5% were unsure (Table 3).

### 3.4. Awareness and Knowledge of Symmetry Options

Overall, only 37.9% of participants reported being aware of the possibility of a symmetry procedure after breast reconstruction. Approximately 40.4% knew that symmetry could involve reduction, augmentation, or lifting. Less than one-third (28.0%) stated that the option of a symmetry procedure had been discussed with them as part of their reconstruction plan. Only 17.5% were aware that health insurance might cover the cost of a symmetry procedure. Similarly, less than one-third (31.2%) knew that symmetry can be performed either simultaneously with reconstruction or at a later stage. Knowledge about potential complications of symmetry procedure was limited, with only 20.4% reporting awareness. In contrast, more than half of participants (57.0%) recognized that achieving symmetry may improve psychological well-being (Table 4).

### 3.5. Attitudes and Perceptions Toward Contralateral Breast Symmetry Procedure

More than half of the participants (53.2%) agreed that they feel comfortable discussing aesthetic concerns with their doctor, while only 12.5% disagreed or strongly disagreed.

The majority considered achieving symmetry important for their body image: 62.4% agreed and 20.1% strongly agreed, with only a small proportion disagreeing (7.0%) or strongly disagreeing (0.3%). Opinions were mixed regarding whether symmetry is necessary after cancer recovery: 31.5% agreed that it is not necessary, while 37.6% disagreed and 14.6% were neutral. Regarding surgical fears, 37.9% agreed they fear the risks of surgery more than the potential benefit, while a similar proportion (38.5%) disagreed. One-third (31.5%) agreed they are considering symmetry to improve their quality of life, while 33.1% disagreed, showing diverse views. Notably, most participants (71.7% agree, 17.5% strongly agree) expressed trust in their medical team’s recommendations. In terms of social impact, 36.3% agreed that body symmetry affects their self-confidence, but 34.7% disagreed. Over half (54.1%) were satisfied with their current breast appearance, but 40.8% still felt uncomfortable with their body after reconstruction. Around 28.0% felt that asymmetry affects their self-image, while 40.4% disagreed. Avoidance of social events due to appearance was relatively low (6.1% agree, 1.6% strongly agree), with over half disagreeing. Finally, about one-quarter (23.9% agree, 5.7% strongly agree) felt that asymmetry causes stress when choosing clothing, while 39.2% disagreed and 22.0% strongly disagreed (Table 5).

### 3.6. Barriers to Symmetry Procedures

**Figure 1:**
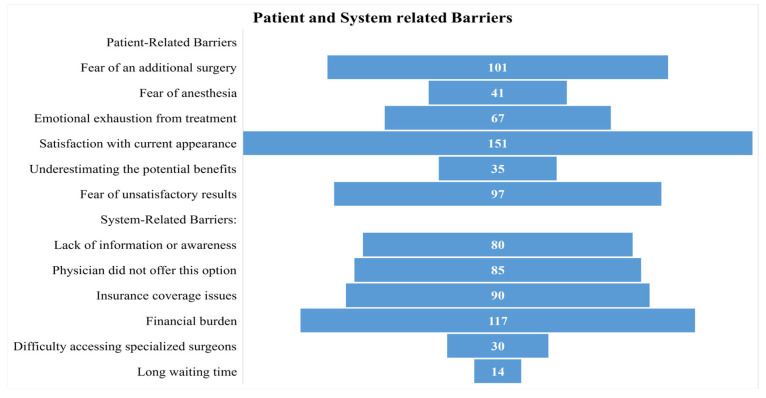
Reported Patient- and System-Related Barriers to Pursuing Symmetry Procedures.

Nearly half of the participants (48.1%) indicated that satisfaction with their current breast appearance was a barrier to considering further symmetry procedures. Fear of undergoing an additional surgery was also common (32.2%), followed by fear of unsatisfactory results (30.9%) and emotional exhaustion from previous treatments (21.3%). Fewer participants reported fear of anesthesia (13.1%) or underestimating the potential benefits of symmetry (11.2%) as barriers. The most commonly reported system-related barrier was the financial burden, cited by over one-third of participants (37.3%). Insurance coverage issues were also noted by 28.7% of respondents, while 27.1% indicated that their physician had not offered a symmetry procedure as an option. One-quarter (25.5%) reported a lack of information or awareness as a barrier to pursuing symmetry. Fewer participants mentioned difficulty accessing specialized surgeons (9.6%) or long waiting times (4.5%) as limiting factors (Figure 1).

## 4. Discussion

In our study, only 6.4% of women who underwent breast reconstruction had previously undergone a contralateral symmetry procedure, and 75% of those women were satisfied with this additional surgery. This finding is very low when compared with the studies from Western countries, which report that the rates of contralateral symmetry procedures after unilateral reconstruction varied from 22% to 67% based on the reconstruction modality and timing. For instance, Losken et al. reported that 67% of the delayed reconstruction patients and 22% of the immediate cases underwent a contralateral symmetry procedure, with higher rates in implant-based reconstructions [19]. One possible cause for the notably lower rate in our setting may be the low level of patient awareness of these symmetry options and, in turn, the suboptimal discussion with their physicians. The awareness of the available procedures among our population was as low as 37.9% and less than a third had discussed them with their doctor. Feeling satisfied with the appearance of their breasts was reported as the main reason not to pursue additional surgery by almost half of the patients, which suggests that patients’ satisfaction with the achieved result hinders the interest in pursuing further surgical intervention. Additionally, many women also expressed fear of more surgery and concerns about unsatisfactory outcomes. System-level barriers such as financial cost and insurance limitations also contributed. Both of these patient and system factors likely contribute to the lower uptake observed in our study and highlight the need for improved education, communication, and financial support to enhance access to contralateral symmetry procedures.

Only 37.9% of our respondents were aware of symmetry options, 28% of whom reported that this option had been discussed. This is much lower than Western studies have reported, where both awareness and discussion have been reported to be above 50% [19]. For example, Retrouvey et al. performed a systematic review and identified lack of patient awareness and poor communication by providers as the two biggest barriers to patients receiving breast reconstruction [20]. Our findings highlight a significant gap in patient education and provider communication, which may contribute to the low uptake of symmetry procedures.

The majority of our participants (62.4%) considered symmetry important for body image, and 75% of those who underwent symmetry procedures were satisfied. This is consistent with previous studies, which report high satisfaction rates (77–82%) among patients who undergo symmetry procedures, particularly when augmentation is performed [14]. On the other hand, in our study, satisfaction with current appearance was a significant obstacle in the pursuit of symmetry, and this was expressed by 48.1% of our participants. This contrasts with studies in Western populations, where dissatisfaction with appearance is more commonly cited as a motivator for symmetry procedures [21]. However, our study uniquely identifies satisfaction with current appearance as a primary barrier, possibly indicating substantial cultural influences specific to Middle Eastern societies, where expectations surrounding feminine identity, modesty, and reconstruction outcomes differ from Western norms [17]. In our context, many women may perceive reconstruction itself as sufficient restoration after cancer treatment, and the pursuit of additional aesthetic perfection may be viewed as less necessary or less culturally emphasized [18]. These cultural dynamics likely shape patient expectations and may partly explain why women in our study felt less compelled to pursue symmetry procedures despite objective asymmetry.

In this study, the barriers included fear of surgery (32.2%) and financial burden (37.3%). These findings are consistent with previous literature, which identifies financial concerns, fear of surgery, and lack of information as key barriers to breast reconstruction and symmetry procedures [22,23,24].

The population in our study was well-educated with urban residency; however, this sociodemographic status was not a factor that led to high rates of undertaking symmetry procedures, suggesting that there is a need to improve awareness and information exchange even among individuals who have a high baseline education and health care access. The predominance of implant-based reconstruction among participants, which is associated with greater difficulty in achieving natural symmetry compared to autologous methods, may have contributed to demand for additional corrective surgery, though this was not reflected in actual procedure rates. The majority of symmetry procedures were in fact done in a delayed setting (55%). This finding reemphasizes the fact that there is still patient reluctance; thus, more intense post-operative education and support is indicated. Patient satisfaction with breast appearance was relatively high, yet a considerable proportion reported discomfort and concerns with body image after reconstruction, highlighting complex and varied psychosocial outcomes among breast cancer survivors. In summary, all of these results highlight the complex impact of clinical factors, timing of surgery, the influence of sociodemographic and psychosocial variables on the decision-making process and outcomes following breast reconstruction.

This study has several limitations. The cross-sectional design and recruitment from a single tertiary cancer center may limit the generalizability of the findings to broader populations or other healthcare settings. Additionally, reliance on patient recall may have introduced recall bias. Although the survey was conducted by trained research personnel using a standardized and validated questionnaire, interviewer bias remains a potential limitation due to the subjective nature of telephone-based responses. Furthermore, the study did not use standardized patient-reported outcome instruments such as the BREAST-Q, which could have provided a more objective and validated assessment of satisfaction with reconstructive and symmetry procedures. Incorporating such tools in future research would strengthen the measurement of patient satisfaction and decision-making processes. Despite these limitations, the study’s strengths include clearly defined inclusion criteria, comprehensive characterization of sociodemographic and clinical variables, and the use of a structured, standardized telephone interview that ensured consistency and reliability in assessing patients’ awareness, attitudes, and perceived barriers toward breast symmetry procedures.

Beyond the above limitations and strengths, it is important to recommend strategies for improving care based on these results. Future research should focus on developing targeted patient education initiatives and enhancing provider communication about symmetry options, as greater awareness is likely to increase uptake and satisfaction with breast reconstruction outcomes. An effective strategy to overcome financial barriers would be the expansion of insurance coverage for symmetry procedures. In addition, future research is necessary to evaluate long-term psychosocial outcomes and procedural trends to address the limitations of cross-sectional studies and provide additional practice-based information. The recommendations above can help to guide breast cancer survivors to optimize breast cancer survivorship and breast symmetry decision-making.

## 5. Conclusions

In this study, the rate of contralateral breast symmetry procedures after reconstruction was much lower than in Western countries, even though symmetry after mastectomy has been shown to be important for quality of life and body image. Patient knowledge, provider communication and education, satisfaction with preexisting appearance, fear of surgery, and cost were found to be the largest contributing factors to the limited access to symmetry procedures. Socio-demographic factors such as high levels of education and urban residence did not translate into improved utilization, underscoring the persistent influence of system-level and informational gaps. Although most women valued symmetry and were very satisfied when they had the procedure, many remained unaware of available options or were deterred by the financial burden or not having discussed with their medical team.

To improve awareness and informed decision-making, targeted strategies are recommended, including structured patient education sessions, culturally sensitive informational materials, incorporation of decision aids in clinical consultations, and proactive discussion by healthcare providers about reconstructive options. Broader financial support and system-level interventions may further facilitate equitable access. Future efforts should also include longitudinal research on psychosocial outcomes and procedural trends to optimize clinical practice and patient-centered care in breast reconstruction.

## Figures and Tables

**Table 1:** Sociodemographic and Clinical Characteristics of the Study Participants.

Characteristic	Overall (N = 314)
Age	
Mean (SD)	45.8 (8.8)
Range	22.0–76.0
Marital status	
Divorced	11 (3.5%)
Married	263 (83.8%)
Single	28 (8.9%)
Widowed	12 (3.8%)
Education	
Primary or less	14 (4.5%)
Secondary	94 (29.9%)
University and higher	206 (65.6%)
Employment status	
No	212 (67.5%)
Yes	102 (32.5%)
Residency	
City	290 (92.4%)
Rural	24 (7.6%)
BMI	
Mean (SD)	28.5 (5.8)
Range	17.7–36.6
Smoking	
Ex-Smoker	36 (11.5%)
Non-Smoker	222 (70.7%)
Active Smoker	56 (17.8%)
Chronic diseases	
No	230 (73.2%)
Yes	84 (26.8%)
Menopausal status	
Postmenopausal	164 (52.2%)
Premenopausal	150 (47.8%)

**Table 2:** Clinical and Treatment Characteristics of the Study Participants.

Variable	Overall (N = 314)
Cancer stage	
I	119 (37.9%)
II	113 (36.0%)
III	82 (26.1%)
Mastectomy type	
Mastectomy	115 (36.6%)
Nipple-sparing mastectomy	40 (12.7%)
Skin-sparing mastectomy	141 (44.9%)
Areola-sparing mastectomy	18 (5.7%)
Type of Reconstruction:	
Autologous tissue	14 (4.5%)
Both	3 (1.0%)
Silicone implant	297 (94.6%)
Time Since Reconstruction (in months)	
Mean (SD)	23.1 (21.9)
Range	1.0–168.0
Radiotherapy	
No	145 (46.2%)
Yes	169 (53.8%)
Chemotherapy	
No	71 (22.6%)
Yes	243 (77.4%)
Hormonal therapy	
No	60 (19.1%)
Yes	254 (80.9%)

**Table 3:** Symmetry Procedures Undertaken and Future Considerations Among Participants.

Question	Overall (N = 314)
Have you undergone a Symmetry procedure?	
No	294 (93.6%)
Yes	20 (6.4%)
If yes, what type?	
Breast Augmentation	8 (40.0%)
Breast Reduction	5 (25.0%)
Mastopexy	7 (35.0%)
Timing of the symmetry procedure:	
Delayed	11 (55.0%)
Immediate	9 (45.0%)
Your level of satisfaction with the result (if done):	
Dissatisfied	5 (25.0%)
Satisfied	15 (75.0%)
If you have not undergone a symmetry procedure: Are you considering it in the future?	
Maybe	69 (23.5%)
No	148 (50.3%)
Yes	77 (26.2%)

**Table 4:** Awareness and Knowledge of Symmetry Procedures Among Participants.

Question	Overall (N = 314)
Aware of symmetry procedure	
No	195 (62.1%)
Yes	119 (37.9%)
Know it includes reduction/augmentation/lift	
No	187 (59.6%)
Yes	127 (40.4%)
Option discussed as part of plan	
No	226 (72.0%)
Yes	88 (28.0%)
Aware insurance may cover it	
No	259 (82.5%)
Yes	55 (17.5%)
Know timing can be same or later	
No	216 (68.8%)
Yes	98 (31.2%)
Aware of possible complications	
No	250 (79.6%)
Yes	64 (20.4%)
Know it may improve psychological well-being	
No	135 (43.0%)
Yes	179 (57.0%)

**Table 5:** Attitudes and Perceptions Toward Symmetry Among Participants.

Question	Overall (N = 314)
I feel comfortable discussing aesthetic concerns with my doctor.	
Agree	167 (53.2%)
Disagree	30 (9.6%)
Neutral	52 (16.6%)
Strongly Agree	52 (16.6%)
Strongly agree	4 (1.3%)
Strongly disagree	9 (2.9%)
Achieving symmetry is important for my body image.	
Agree	196 (62.4%)
Disagree	22 (7.0%)
Neutral	28 (8.9%)
Strongly agree	63 (20.1%)
Strongly disagree	1 (0.3%)
Strongly agree	4 (1.3%)
I do not believe that symmetry is necessary after recovering from cancer.	
Agree	99 (31.5%)
Disagree	118 (37.6%)
Neutral	46 (14.6%)
Strongly agree	23 (7.3%)
Strongly disagree	28 (8.9%)
I fear the risks of surgery more than the potential aesthetic benefit.	
Agree	119 (37.9%)
Disagree	121 (38.5%)
Neutral	34 (10.8%)
Strongly agree	20 (6.4%)
Strongly disagree	20 (6.4%)
I am considering symmetry to improve my quality of life.	
Agree	99 (31.5%)
Disagree	104 (33.1%)
Neutral	55 (17.5%)
Strongly agree	35 (11.1%)
Strongly disagree	21 (6.7%)
I trust my medical team to recommend what is best for me.	
Agree	225 (71.7%)
Disagree	8 (2.5%)
Neutral	25 (8.0%)
Strongly agree	55 (17.5%)
Strongly disagree	1 (0.3%)
Body symmetry affects my self-confidence in social settings.	
Agree	114 (36.3%)
Disagree	109 (34.7%)
Neutral	29 (9.2%)
Strongly agree	23 (7.3%)
Strongly disagree	39 (12.4%)
I am satisfied with the current appearance of my breast.	
Agree	170 (54.1%)
Disagree	54 (17.2%)
Neutral	28 (8.9%)
Strongly agree	49 (15.6%)
Strongly disagree	13 (4.1%)
Do you feel uncomfortable with your body after reconstruction?	
Agree	128 (40.8%)
Disagree	95 (30.3%)
Neutral	31 (9.9%)
Strongly agree	26 (8.3%)
Strongly disagree	34 (10.8%)
Does asymmetry affect your self-image?	
Agree	88 (28.0%)
Disagree	127 (40.4%)
Neutral	37 (11.8%)
Strongly agree	9 (2.9%)
Strongly disagree	53 (16.9%)
Do you avoid social events because of your appearance?	
Agree	19 (6.1%)
Disagree	169 (53.8%)
Neutral	14 (4.5%)
Strongly agree	5 (1.6%)
Strongly disagree	107 (34.1%)
Does asymmetry cause stress when choosing clothing?	
Agree	75 (23.9%)
Disagree	123 (39.2%)
Neutral	29 (9.2%)
Strongly agree	18 (5.7%)
Strongly disagree	69 (22.0%)

## Data Availability

The data that support the findings of this study are available from the corresponding author upon reasonable request. The data are not publicly available due to privacy and ethical restrictions.
